# Blood biomarker profiles in autopsy‐diagnosed carriers of different PSEN1 mutations relative to sporadic Alzheimer’s disease and controls

**DOI:** 10.1002/alz.092528

**Published:** 2025-01-09

**Authors:** Tara K Lafferty, Anuradha Sehrawat, Xuemei Zeng, Yijun Chen, William E Klunk, Tharick A. Pascoal, Ann D Cohen, Victor L Villemagne, M. Ilyas Kamboh, Oscar L. Lopez, Eric E Abrahamson, Sarah Berman, Julia Kofler, Milos D Ikonomovic, Thomas K Karikari

**Affiliations:** ^1^ University of Pittsburgh, Pittsburgh, PA USA; ^2^ University of Pittsburgh Alzheimer's Disease Research Center (ADRC), Pittsburgh, PA USA; ^3^ University of Pittsburgh Alzheimer's Disease Research Center, Pittsburgh, PA USA; ^4^ VA Pittsburgh Healthcare System, Pittsburgh, PA USA

## Abstract

**Background:**

Specific PSEN1 mutations cause early‐onset AD but their effects on blood biomarker levels are unknown. We evaluated autopsy‐confirmed individuals affected by six different PSEN1 mutations; two of known (L381V, C410Y) and three (A426P/E318G, M233L, and V261I) of unknown pathogenic status. The sixth patient had Autosomal Dominant AD (ADAD) not yet genotyped. Neuropathologically diagnosed sporadic AD (sAD; n=8) and unaffected controls (n=7) were included for comparison.

**Method:**

The participants, except the controls, had brain autopsy performed under informed consent at the University of Pittsburgh Alzheimer’s Disease Research Center. Genetic testing was performed to confirm the presence of PSEN1 mutation(s). Blood was collected, processed, and stored during life. Plasma p‐tau217, p‐tau181, neurofilament light (NfL), glial fibrillary acidic protein (GFAP), amyloid beta (Ab) 40 and 42 were measured using commercial assays on Simoa HDX at the Biofluid Biomarker Laboratory, Department of Psychiatry, University of Pittsburgh.

**Result:**

Plasma p‐tau217 and p‐tau181 were increased 5‐8‐fold and 3‐4‐fold in the PSEN1 and sAD groups respectively versus controls. GFAP had 4‐fold increase in both sAD and PSEN1 groups while NfL was highest in sAD. Ab42, Ab40 and Ab42/40 showed no between‐group differences. Individuals with the two pathogenic mutations recorded the highest levels of p‐tau217, p‐tau181, GFAP and NfL, with the A426P/E318G carrier also having a comparably high p‐tau181.

**Conclusion:**

Marked elevation of plasma p‐tau and GFAP suggest increased tau phosphorylation and astrogliosis in ADAD while neurodegeneration intensity (by NfL) may be comparable to sAD.

